# Acute-on-Chronic Liver Failure in Cirrhosis

**DOI:** 10.3390/jcm10194406

**Published:** 2021-09-26

**Authors:** Carmine Gambino, Salvatore Piano, Paolo Angeli

**Affiliations:** Unit of Internal Medicine and Hepatology (UIMH), Department of Medicine (DIMED), University of Padova, 35128 Padova, Italy; carmine.gabriele.gambino@gmail.com (C.G.); pangeli@unipd.it (P.A.)

**Keywords:** acute-on-chronic liver failure (ACLF), cirrhosis, liver transplantation (LT)

## Abstract

Acute-on-chronic liver failure (ACLF) is a syndrome that develops in patients with acutely decompensated chronic liver disease. It is characterised by high 28-day mortality, the presence of one or more organ failures (OFs) and a variable but severe grade of systemic inflammation. Despite the peculiarity of each one, every definition proposed for ACLF recognizes it as a proper clinical entity. In this paper, we provide an overview of the diagnostic criteria proposed by the different scientific societies and the clinical characteristics of the syndrome. Established and experimental treatments are also described. Among the former, the most relevant are directed to support organ failures, treat precipitating factors and carry out early assessment for liver transplantation (LT). Further studies are needed to better clarify pathophysiology of the syndrome and discover new therapies.

## 1. Definition of Acute-on-Chronic Liver Failure

Acute decompensation (AD) of cirrhosis refers to the development of ascites, gastrointestinal haemorrhage, hepatic encephalopathy or any combination of these, which leads to hospital admission [1]. Acute-on-chronic liver failure (ACLF) is a distinct syndrome that develops in patients with acutely decompensated chronic liver disease and is characterised by high 28-day mortality. Other major features of ACLF are the strong association with one or more precipitating factor(s), the development of single- or multiple organ failures (OFs) and a severe degree of systemic inflammation [2,3,4]. International scientific societies have proposed different definitions of ACLF in recent years; they differ from each other mainly in the type of precipitant (hepatic or extrahepatic), the stage of underlying liver disease (chronic hepatitis or cirrhosis) and the inclusion or not of extra-hepatic OFs. In spite of these differences, each of them recognizes ACLF as a definite clinical entity. Table 1 summarizes definitions, diagnostic criteria and stratification of ACLF used by the four major international consortia [2,5,6,7,8,9].

The definition proposed by the European Association for the Study of the Liver—Chronic Liver Failure (EASL-CLIF) Consortium is based on the results of the CANONIC study, a multi-center prospective investigation in which 1343 patients non-electively hospitalized for AD of cirrhosis were enrolled, irrespective of prior episode(s) of AD [2]. This definition considers both hepatic and extra-hepatic precipitants and both liver and extra-hepatic OFs. The diagnosis of OFs is based on a modified Sequential Organ Failure Assessment (SOFA) score, called CLIF-C organ failure (CLIF-C OF), which considers the function of six organ systems (liver, kidney, brain, coagulation, circulation and respiration) [2]. According to the number of OFs, patients with ACLF were stratified into three groups: (I) patients with a single kidney failure or another single OF if associated with brain or kidney disfunction (ACLF grade 1); (II) patients with two OFs (ACLF grade 2); (III) patients with three or more OFs (ACLF grade 3) [2]. We contributed to the development of this definition, which nowadays is the most studied. Thus, we currently use it in our center.

The definition proposed by the North American Consortium for the Study of End-stage Liver Disease (NACSELD) is based on an investigation involving 507 patients with AD of cirrhosis non electively hospitalised for infection [5]. Like the European one, the North American definition considers extra-hepatic OFs as part of the syndrome but does not include liver and coagulation. It defines ACLF by the presence of two or more OFs among kidney, brain, circulation and respiration and stratifies patients according to the number of organ failures [5]. The Chinese Group on the Study of Severe Hepatitis B (COSSH) developed a definition for hepatitis B virus (HBV)-related ACLF by using data from a large cohort of 1202 patients with HBV-related AD, with or without cirrhosis. The CLIF-C OF scoring system was used to define OFs; so, this definition and the consequent stratification of patients are quite similar to the European ones. The only difference is that, in the Chinese classification, a patient with single liver failure with INR ≥ 1.5 is considered as having ACLF grade 1 [6]. The Asian Pacific Association for the Study of the Liver (APASL) proposed a definition of ACLF in 2009 which was based on an expert opinion. This definition was updated by the APASL ACLF Research Consortium (AARC) in 2014 and then in 2019, using the results of the AARC database (5228 patients collected at that time) [7,8,9]. Unlike the above definitions, AARC investigators consider extra-hepatic OFs as manifestations but not as components of the syndrome, and extra-hepatic insults (for example, bacterial infections) as complications, but not triggers, of ACLF. So, ACLF is considered as an acute hepatic insult (for example, HBV reactivation or acute alcoholic hepatitis), manifested as jaundice (total bilirubin levels ≥ 5 mg/dL) and coagulation failure (INR ≥ 1.5 or prothrombin activity < 40%) and complicated by clinical ascites, encephalopathy or both within 4 weeks in patients with chronic liver disease or compensated cirrhosis without prior decompensation and with no AD [9]. Thus, AARC investigators consider ACLF to be totally distinct from acutely decompensated cirrhosis. The severity of ACLF is assessed using a grading system based on the AARC score [9].

## 2. Clinical Features

ACLF has typical clinical features based on the definition used, on which its prevalence also depends. In the European cohort, the prevalence of the syndrome was 23% among patients with AD of cirrhosis at admission and 8.3% of patients developed it during hospitalization within a period of days (maximum of two weeks). In outpatients with cirrhosis, the incidence of the syndrome is about 40% at 10 years [11]. As confirmed by the PREDICT (PREDICTing Acute-on-Chronic Liver Failure) study, another large-scale European prospective investigation designed to identify predictors of this syndrome, patients with ACLF were younger, showed higher levels of white blood cells and C-reactive protein (CRP) and had a greater prevalence of bacterial infections, severe alcoholic hepatitis, variceal bleeding, drug-induced encephalopathy as precipitants, with respect to patients without ACLF [2,12,13]. Moreover, the PREDICT study demonstrated that the clinical course of AD that leads to ACLF is distinct from the other forms of AD of cirrhosis [12]. The 28-day mortality significantly rises with the increase in the number of OFs, ranging from 4.7% for patients without ACLF to 22%, 32% and 77% for patients with ACLF grade 1, 2 and 3, respectively [2].

In a validation study of NACSELD definition of ACLF, in which 2675 patients with AD of cirrhosis related or not to infection were included, the prevalence of ACLF was 10% and 30-day mortality rate was significantly different between patients with or without the syndrome (41% vs. 7%, respectively) [14]. In a recent study, the NACSELD criteria were demonstrated to be less sensitive compared to EASL-CLIF criteria in diagnosing ACLF [15].

When using NACSELD criteria, only about 40% of patients with a diagnosis of ACLF based on EASL-CLIF criteria were classified as affected by the syndrome, probably because the NACSELD definition considers only more severe patients and because it could be influenced by the medical strategies available in the different centers (renal replacement therapy (RRT), mechanical ventilation, use of vasopressors) [10,16].

In a cohort of patients with HBV-related AD of cirrhosis, the prevalence of ACLF was 30.2% according to the COSSH ACLF definition. As in the European cohort, patients with ACLF were younger, had a more severe grade of systemic inflammation (as demonstrated by higher levels of white blood cells and CRP) and more frequently had a bacterial infection (associated or not with HBV reactivation) as precipitant compared to those without ACLF, with a significantly higher short-term mortality (52.1% vs. 4.3%) [6]. Although EASL-CLIF and COSSH definitions of ACLF are very similar, clinical characteristics of patients are quite different, because of the higher prevalence of intra-hepatic precipitants in the Chinese cohort (most often HBV reactivation) with respect to the European cohort [16], with liver and coagulation failure being more frequent in the former and kidney and brain failure more frequent in the latter [4]. As expected, a flare of HBV infection was the most frequent trigger of ACLF in studies using AARC criteria [9,17]. In a study using AARC ACLF criteria which enrolled patients with HBV-related ACLF, about 32% had a bacterial or fungal infection as a complication. The 28-day mortality rate was 27.8% [18].

## 3. Pathophysiology

**Figure 1:**
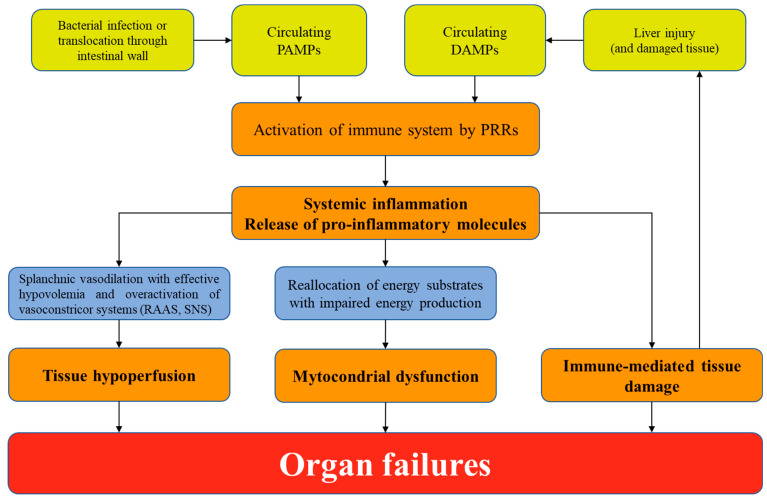
Pathophysiology of ACLF. PAMPs, pathogen-associated molecular patterns; DAMPs, damage-associated molecular patterns; PRR, pattern-recognition receptors; RAAS, renin-angiotensin-aldosterone system; SNS, sympathetic nervous system.

The pathophysiology of ACLF is yet to be fully understood. To date, ACLF is considered the extreme expression of systemic inflammation that drives AD of cirrhosis [19]. Systemic inflammation is characterised by activation of the immune system that leads to increased circulating levels of inflammatory mediators and, if severe, proliferation of neutrophils, monocytes and dendritic cells [20]. The mechanism of systemic inflammation depends on the precipitant of ACLF [3]. The recognition of pathogen-associated molecular patterns (PAMPs) activates the innate immune system by pattern-recognition receptors (PRR) in case of bacterial infection or translocation of viable bacteria and bacterial products through the intestinal wall [19,21]. Exceeding inflammation can cause direct tissue damage and necrotic cell death, resulting in the release of damage-associated molecular patterns (DAMPs) that perpetuate inflammation acting on PRR [21]. DAMPs are also released when an injury acts directly on the liver, as in case of alcoholic hepatitis or ischemia due to variceal haemorrhage [22,23]. This overactivation of the immune cells requires a large amount of energy sustained by reallocation of nutrients. This causes a reduced availability of substrates for other organ systems that leads to OFs by severe mitochondrial dysfunction and impaired energy production [19]. Moreover, recent findings suggest that systemic inflammation can explain and act with the traditionally accepted organ-specific mechanisms of AD (portal hypertension, hyperammonaemia, endogenous vasoconstrictors system and arterial blood volume) in determining OFs [19]. Blood metabolomics offers a new insight into the pathophysiology of systemic inflammation in patients with ACLF and could be an intriguing starting point to uncover new potential therapeutic targets [24]. Figure 1 summarizes the pathophysiology of ACLF.

## 4. Prognostic Stratification

ACLF is a dynamic syndrome that can resolve, improve or worsen in a few days [25]. Outcomes for ACLF patients are strictly related both to severity of liver disease and to severity and number of OFs. Because ACLF patients may be considered for urgent Intensive Care Unit (ICU) referral and/or liver transplantation (LT), different consortia developed prognostic scores [16]. EASL-CLIF proposed the CLIF-C ACLF score, which demonstrated more accuracy in predicting death than MELD (Model for end-stage Liver Disease), MELD-Na (Model for end-stage Liver Disease-Sodium), Child-Pugh and CLIF-C OF scores [17]. CLIF-C ACLF score captures both intra- and extra-hepatic OFs but has a subjective element in the scoring of hepatic encephalopathy and a “ceiling effect” with INR, serum creatinine and bilirubin (for example, a patient with serum bilirubin 25 mg/dL has the same prognosis of a patient with serum bilirubin 12 mg/dL) [16]. The NACSELD organ failure score is simple to use but considers only the sickest patients. The AARC-ACLF score was found to be superior to MELD and CLIF-SOFA in predicting short-term mortality [26] but, as with CLIF-C ACLF score, has subjective elements and suffers from a “ceiling effect” for the considered laboratory values [16]. The COSSH-ACLF score showed higher predictive value for short-term mortality than other scores (MELD, MELD-Na, Child-Pugh, CLIF-C OF and CLIF-C ACLF) in patients with HBV-ACLF [6]. Recently, a simplified version of this score (COSSH-ACLF II) demonstrably improved prognostic accuracy and sensitivity for patients with HBV-ACLF. The COSSH-ACLF II score also allows easy division of patients into three different strata with significantly different 28-day mortality rates [27]. The COSSH-ACLF scores also include a subjective element in hepatic encephalopathy evaluation.

Prognosis is more accurately estimated when the scores are applied at 3 to 7 days than at time of diagnosis [25,28]. These findings are in keeping with the dynamic nature of ACLF. Prognostic scores have also been applied to determine futility of treatments in ACLF patients [28,29]. Thus, it is necessary to overcome the above-mentioned limitations by creating models based only on objective, verifiable and continuous variables [16]. Finally, among OFs not actually included in the prognostic scores, relative adrenal insufficiency (RAI) has been shown to have a similar prognostic value for non-kidney OFs. RAI could be considered to better stratify patients with ACLF in clinical practice [30].

## 5. Management of ACLF

Principles of treatment of ACLF are summarized in Table 2.

### 5.1. Admission to Intensive Care Unit

The referral of patients with ACLF to ICU should be neither delayed nor denied only because of the underlying chronic liver disease or the possibility of poor prognosis in patients with OF(s) [31,32]. In fact, several findings suggest that acceptable survival rates can be achieved in patients with cirrhosis admitted to ICU [33]. In such a setting, CLIF-C OF and CLIF-C ACLF scores perform better than generally used and liver specific scores [31,34].

**Table 2:** Principles of treatment of ACLF [3]. ICA, International Club of Ascites; AKI, acute kidney injury; HRS, hepatorenal syndrome; RRT, renal replacement therapy; LT, liver transplantation; NSAID, non-steroidal anti-inflammatory drugs; MAP, mean arterial pressure; SBP, spontaneous bacterial peritonitis; LVP, large volume paracentesis; DVT deep-vein thrombosis; PaO_2_ FiO_2_ SpO_2_ ACLF, acute-on-chronic liver failure.

Kidney	Circulation	Coagulation	Lung	Brain	Infections
Assess AKI severity using ICA Criteria * Taper/withdraw from diuretics and beta-blockers, withdraw from nephrotoxic drugs	Assess hemodynamic state early; consider a MAP ≥ 65 mmHg as target	Assess complete blood count and coagulation tests	Assess respiratory state by using also imaging techniques Calculate PaO_2_/FiO_2_ or SpO_2_/FiO_2_	Assess hepatic encephalopathy using West Haven criteria. Identify and treat the underlying cause	Perform a complete work up for infection at ACLF diagnosis
Administer albumin (1 g/kg for 48 h) if AKI stage > 1a * to volume expansion; if HRS-AKI, administer terlipressin by continuos infusion (2 mg/24 h) and albumin (20/40 g/day)	Administer crystalloids and 5% albumin as resuscitation fluids; norephinephrine as first line vasopressor	Administer platelets (if < 20.000 × 10^9^/L) and fibrinogen (if <1 g/L) if invasive procedures	Administer oxygen and ventilation with lung protective strategy	Administer lactulose and enemas for hepatic encephalopathy.	Administer broad spectrum high-dose antibiotics at ACLF diagnosis and frequently re-assess therapy
Consider RRT as bridge to LT	Consider 20% albumin if AKI (see Kidney), SBP, LVP; consider terlipressin if additional agent needed	Consider prophylaxis for DVT in patients without severe coagulopathy	Consider intubation if risk of aspiration (West Haven grade III or IV hepatic encephalopathy)	Consider short-acting sedative agents if necessary	Consider antifungal agents if risk factors for fungal infections
Avoid NSAIDs	Avoid starches	Avoid fresh frozen plasma to correct INR if no bleeding	Avoid delay in intubation even if normal blood oxygen level	Avoid deep sedation and benzodiazepines	Avoid delay in antibiotics administration

* See ref. [34].

### 5.2. Treating Organ Failures

Acute kidney injury (AKI) should be treated with volume expansion with albumin and withdraw from diuretics and beta-blockers [35]. If there is no response after two days of volume expansion and hepatorenal syndrome (HRS)-AKI criteria are met [36], terlipressin given by continuous infusion should be started [37]. Response to terlipressin is inversely related to the number of OFs at baseline and to the creatinine value at the start of the treatment [38,39]. There are scarce data about the role of RRT in patients with ACLF. In a recent study in patients with type 1 HRS and no response to vasoconstrictors, RRT did not improve survival at 30 and 180 days [40]. To date, RRT should be considered as a bridge to LT in selected patients. A target of mean arterial pressure ≥ 65 mmHg should be reached within the first hours in patients with circulatory failure. Crystalloids and 5% albumin solution should be preferred over saline solutions as resuscitation fluids. Starches formulations should be avoided [4]. Norephinephrine is the first-line vasopressor agent [41]. Terlipressin demonstrated a better alternative in one study in patients with cirrhosis and septic shock [42]. Infusion of blood products should be considered only if clinically significant bleeding or invasive procedures in patients with coagulation failure. Respiratory failure should be treated with oxygen supplementation and ventilation, if needed. Intubation should be considered to prevent aspiration pneumonia in patients with severe hepatic encephalopathy by using short-acting sedative agents. Other measures include lactulose and enemas to clear the bowel and the treatment of the underlying cause [4,35].

## 6. Treating the Precipitating Event

### 6.1. Bacterial or Fungal Infection

The prevalence of infections in patients with ACLF, either as precipitants or complications of the syndrome, is about 50% and rises to 70% in patients with three or more OFs [43]. Bacterial infections are more frequent than fungal ones, being multidrug-resistant (MDR) pathogens involved in one-third of cases with different prevalence related to geographical region [43,44]. A complete work up for infection, including microbiological and imaging examinations, should be performed in all patients at diagnosis of ACLF before starting high-dose broad-spectrum antimicrobial therapy. The broad spectrum antibiotic treatment should be started as soon as possible. An effective antibiotic treatment is strongly associated with an improvement in survival in patients with ACLF [45,46]. Antifungal agents should be considered in patients with risk factors for fungal infections (e.g., nosocomial infections, previous antibiotic treatment, diabetes, AKI, recent endoscopy) [47,48].

### 6.2. Alcoholic Hepatitis

Corticosteroids are the first-line treatment for severe alcoholic hepatitis. The Lille score is used to identify response to treatment. The probability of response to corticosteroids is lower in patients with ACLF respect to those without (38% and 77%, respectively) and is negatively correlated with the number of OFs at diagnosis [49].

### 6.3. Acute Variceal Haemorrhage

Standard medical treatment for this life-threatening precipitant is made by a vasoconstrictor (terlipressin, somatostatin or analogues such as octreotide) and endoscopic therapy (preferably variceal ligation) plus a short-term antibiotic prophylaxis with ceftriaxone [50]. In a recent multicenter international study which enrolled patients with acute variceal bleeding and ACLF, the syndrome was identified as an independent risk factor for rebleeding and short-term mortality. Pre-emptive TIPS may improve survival in this cluster of patients, but further studies are needed before recommending its routinary use [51].

### 6.4. Hepatitis B Virus Reactivation

All patients with hepatitis B virus infection at presentation should be treated with a nucleoside or nucleotide analogue. Tenofovir, tenofovir alafenamide or entecavir should be used [9].

### 6.5. Liver Transplantation

Several studies showed that LT improved survival in patients with ACLF [52,53]. In a recent multi-center European investigation, one-year post-LT survival was >than 80% independently from ACLF grade [54]. Despite these findings, prioritization for LT of patients with ACLF remains complicated. Commonly used scores for listing patients with cirrhosis were demonstrated not to be accurate enough to predict survival in patients with OFs. Mortality of patients with ACLF of grade 3 and a MELD score < 25 was shown to be higher than in patients with a MELD score > 35 but without ACLF [52]. MELD-Na score underestimates mortality at 90-days in patients with ACLF, especially in those with MELD-Na < 30 [55]. Moreover, patients with ACLF grade 3 had a greater waitlist 14-day mortality than patients listed as status 1a, independent of MELD-Na score [56]. These findings emphasize the importance of early discussion for LT and consideration of priority for patients with ACLF, irrespective of traditional listing scores. Recently, a novel score which incorporates MELD score and ACLF grade demonstrably performs better than traditional scores by giving a higher impact to ACLF grade at lower MELD listing [57].

The Spanish Society of Liver Transplantation (SETH) proposed a consensus statement in which expedited organ allocation is recommended to allow ACLF patients to be transplanted [58]. SETH recommends the use of CLIF-C ACLF score instead of MELD to assess prognosis and suggests prioritisation of these patients because of their poor short-term prognosis [58]. NHS Blood and Transplant recently set the ACLF Liver Transplantation Tier (ACLFLTT) which gives a priority below that of super-urgent listed patients to those with cirrhosis and liver failure (as manifested by jaundice and coagulopathy) who stay on ICU for organ support and have risk of 28-day mortality of >50%. These patients usually fulfill EASL-CLIF criteria for ACLF of grade 2 or 3 [59].

An optimal selection of candidates for LT is equally important to avoid futile LT. Factors independently associated with poor post-LT survival were found to be lactate levels > 4 mmol/L, need for RRT at LT, older age of recipient, use of marginal organs and infections with MDROs while on the waiting list [52,54,60].

### 6.6. Extracorporeal Liver Support

Two large randomized clinical trials demonstrated no improvement in short-term survival in ACLF patients treated with albumin dialysis versus standard medical therapy [61,62]. Other two randomized trials are currently assessing plasma exchange (APACHE trial; ClinicalTrials.gov number, NCT03702920) and albumin exchange with endotoxin removal (DIALIVE trial, NCT03065699).

### 6.7. Granulocyte-Colony Stimulating Factor

Two small single-center studies reported improved survival and reduced rate of bacterial infections in ACLF patients treated with Granulocyte-Colony Stimulating Factor (G-CSF) [63,64]. This result was not confirmed by the recent large multicenter randomized trial (GRAFT study), which failed to demonstrate the superiority of G-CSF over standard medical treatment and reported serious drug-related adverse events [65].

### 6.8. Human Allogeneic Liver-Derived Progenitor Cells

Low doses of human allogeneic liver-derived progenitor cells (HALPC) appeared to be safe in a clinical phase II study which involved 24 patients [66]. Further studies are needed to confirm safety and assess efficacy of this medicinal product.

## 7. Conclusions

ACLF is a distinct syndrome without a universally accepted definition and is characterized by high short-term mortality due to OFs. Patients with ACLF should access ICU without delay if necessary. LT has good outcomes and should be considered irrespective of traditionally used scores for waiting list allocation. Prioritization of ACLF for LT should be improved using proper scores for ACLF patients. Further studies are needed in order to better clarify the pathophysiology of the syndrome and to develop treatments other than supportive measures for OFs.

## Figures and Tables

**Table 1:** Definitions, diagnostic criteria, and stratification of ACLF used by the four major international consortia *.

Characteristics	EASL-CLIF Consortium	NACSELD	COSSH	AARC
Population	Patients with AD of cirrhosis, independently from the absence/presence of previous AD	Patients with AD of cirrhosis, independently from the absence/presence of previous AD	Patients with AD of HBV-related chronic liver disease, with or without cirrhosis	Patients with CLD or compensated cirrhosis and acute liver insult that causes acute liver deterioration
Precipitating events	Intrahepatic (alcoholic hepatitis), extrahepatic (infection, gastrointestinal bleeding), or both	Intrahepatic, extrahepatic, or both	Intrahepatic (HBV flare), extrahepatic (bacterial infection) or both	Intrahepatic
Criteria of organ system failures used to define ACLF	Liver: Total bilirubin ≥ 12 mg/dL; Kidney: Creatinine ≥ 2 mg/dL or use of RRT; Coagulation: INR ≥ 2.5; Brain: HE Grade 3–4 in West Haven classification or use of mechanical ventilation because of HE; Circulation: Use of vasopressors including terlipressin; Lung: PaO_2_/FiO_2_ ≤ 200 or SpO_2_/FiO_2_ ≤ 214, or use of mechanical ventilation for reason other than HE	Kidney: Use of dialysis or other form of RRT; Brain: HE Grade 3–4 in West Haven classification; Circulation: MAP <60 mmHg or reduction of 40 mmHg in SBP from baseline, in spite of fluid resuscitation and adequate cardiac output; Lung: Use of mechanical ventilation	Same criteria as those used by the EASL-CLIF Consortium	Liver: Total bilirubin levels ≥ 5 mg/dL Brain: clinical HE
Criteria for the presence of ACLF and ACLF stratification	ACLF is stratified into 3 grades of increasing severity. - ACLF grade 1 contains 3 subgroups of patients with: (1) single kidney failure (2) single liver, coagulation, circulatory or lung failure that is associated with either kidney dysfunction, brain dysfunction, ^a^ or both; (3) single brain failure and kidney dysfunction ^a^; - ACLF grade 2: two OFs; - ACLF grade 3: three or more OFs.	Patients are stratified according to the number of organ failures (2, 3, or 4 organ failures)	ACLF is stratified into 3 grades of increasing severity. - ACLF grade 1 contains 4 subgroups of patients with: (1) single kidney failure; (2) single liver failure and either INR ≥ 1.5, kidney dysfunction, brain dysfunction, ^a^ or any combination of these; (3) single coagulation, circulatory or respiratory failure and either kidney dysfunction, brain dysfunction, ^a^ or both; (4) cerebral failure alone and kidney dysfunction; - ACLF grade 2: two OFs - ACLF grade 3: three or more OFs	Total bilirubin levels of 5 mg/dL or more and INR ≥ 1.5 or prothrombin activity <40% complicated within 4 weeks byclinical ascites, HE, or both. The severity of ACLF is assessed using the AARC score ^#^: Grade 1 by scores 5–7, Grade 2 by scores 8–10 and Grade 3 for 11–15.
Short-term mortality rate of ACLF	By 28 days: Grade 1: 22% Grade 2: 32% Grade 3: 77%	By 30 days: 2 organ failures: 49% 3 organ failures: 64% 4 organ failures: 77%	By 28 days: Grade 1: 23% Grade 2: 61% Grade 3: 93%	By 30 days: Grade 1: 13% Grade 2: 45% Grade 3: 86%

EASL-CLIF, European Association for the Study of the Liver—Chronic Liver Failure; NACSELD, North American Consortium for the Study of End-stage Liver Disease; COSSH, Chinese Group on the Study of Severe Hepatitis B; AARC, APASL ACLF Research Consortium; APASL, Asian Pacific Association for the Study of the Liver; AD, acute decompensation; CLD, chronic liver disease ACLF, acute-on-chronic liver failure; RRT, renal replacement therapy; HE, hepatic encephalopathy; OFs, organ failures; INR, international normalised ratio; MAP, mean arterial pressure; SBP, systolic blood pressure. * Adapted from ref. [10]; ^#^ See ref. [9]. ^a^ Kidney dysfunction: serum creatinine from 1.5 mg/dL to 1.9 mg/dL. Brain dysfunction: grade 1 or grade 2 HE.
